# Crystal structure and Hirshfeld surface analysis of 3,3′-(sulfanedi­yl)bis­(2-iodo-1-methyl-1*H*-indole)

**DOI:** 10.1107/S2056989026002872

**Published:** 2026-03-27

**Authors:** Adisa Ayobami, Abid Shaikh, Clifford W. Padgett

**Affiliations:** a Georgia Southern University, 521 College of Education Dr Department of Chemistry Biochemistry and Physics Statesboro GA 30458 USA; b Georgia Southern University, 11935 Abercorn St Department of Chemistry Biochemistry and Physics Savannah GA 31419 USA; Universidade Federal do ABC, Brazil

**Keywords:** bis­(indol­yl)sulfane, synthesis, crystal structure

## Abstract

The title compound, C_18_H_14_I_2_N_2_S, comprises two *N*-methyl-2-iodo­indole fragments linked at the 3-positions by a thio­ether bridge. The sulfur atom adopts a typical thio­ether geometry, and the C—S—C linkage is *gauche* on both sides, with C—S—C—C torsion angles of −64.1 (11) and −48.3 (10)°. The indole units are essentially planar and are strongly inclined to one another, with an inter­planar angle of 103.3 (3)°. The compound crystallizes in the ortho­rhom­bic space group *Fdd2*; the mol­ecule lacks classical hydrogen-bond donors, and no significant hydrogen bonding or π–π stacking is observed. The packing is dominated by van der Waals inter­actions together with short I⋯C and I⋯π contacts, giving a herringbone arrangement and forming chains along the *b* axis. Hirshfeld surface analysis indicates that H⋯H (33.1%), H⋯C/C⋯H (25.5%) and H⋯I/I⋯H (24.4%) contacts make the largest contributions to the crystal packing.

## Abstract 2

The title compound consists of two *N*-methyl-2-iodo­indole units linked by a thio­ether bridge in a *gauche* conformation. In the crystal, short I⋯C and I⋯π contacts dominate the packing and generate a herringbone motif.

## 1. Chemical context

Sulfur-bridged indolyl compounds have attracted inter­est because of their relevance to medicinal chemistry (Xalxo *et al.*, 2023 ▸; Silveira *et al.*, 2013 ▸) and materials science (Yuan *et al.*, 2022 ▸), as well as their ability to serve as versatile synthons in organic chemistry (Wang, 2024 ▸). Di­aryl­ated thio­ethers (ar­yl–S–aryl motifs) are used in functional materials; for example, thio­ether-linked polymeric systems have been developed for adsorption/capture applications such as iodine uptake (Shetty *et al.*, 2022 ▸), and porous poly(aryl thio­ether) frameworks have also been explored for metal capture, sensing, and heterogeneous catalysis (Rivero-Crespo *et al.*, 2021 ▸). Thieno­indole-based π-systems have also been incorporated into conjugated polymers for organic electronic applications (Jeong *et al.*, 2016 ▸). The motivation for studying these motifs comes from the central role of indole scaffolds in drug discovery (Mo *et al.*, 2024 ▸) and from literature reports that indole-based aryl sulfides can exhibit potent anti­bacterial activity against *Staphylococcus aureus* (Lavekar *et al.*, 2024 ▸). Organosulfur compounds containing thio­ether (sulfane) linkages are known to influence mol­ecular conformation, electronic properties, and inter­molecular inter­actions in the solid state (Gundermann, 1963 ▸). Incorporation of a sulfide bridge between two indole moieties can enhance structural rigidity while allowing conformational flexibility about the C–S–C linkage (Mohanty *et al.*, 2025 ▸). The presence of iodine atoms at the 2-position of the indole rings provides opportunities for specific inter­molecular contacts, while *N*-methyl­ation suppresses classical N—H hydrogen bonding, allowing a clearer assessment of the roles played by halogen⋯halogen, halogen⋯π, and sulfur-involved inter­actions in the crystal structure (Bergman & Janosik, 2002 ▸). 3,3′-Sulfanediylbis(2-iodo-1-methyl-1*H*-indole) serves as a model system for assessing I⋯I, I⋯π and sulfur-involving contacts in the absence of classical hydrogen-bond donors, providing guidance for the crystal engineering of closely related derivatives. Herein, we report its synthesis and single-crystal X-ray diffraction analysis.
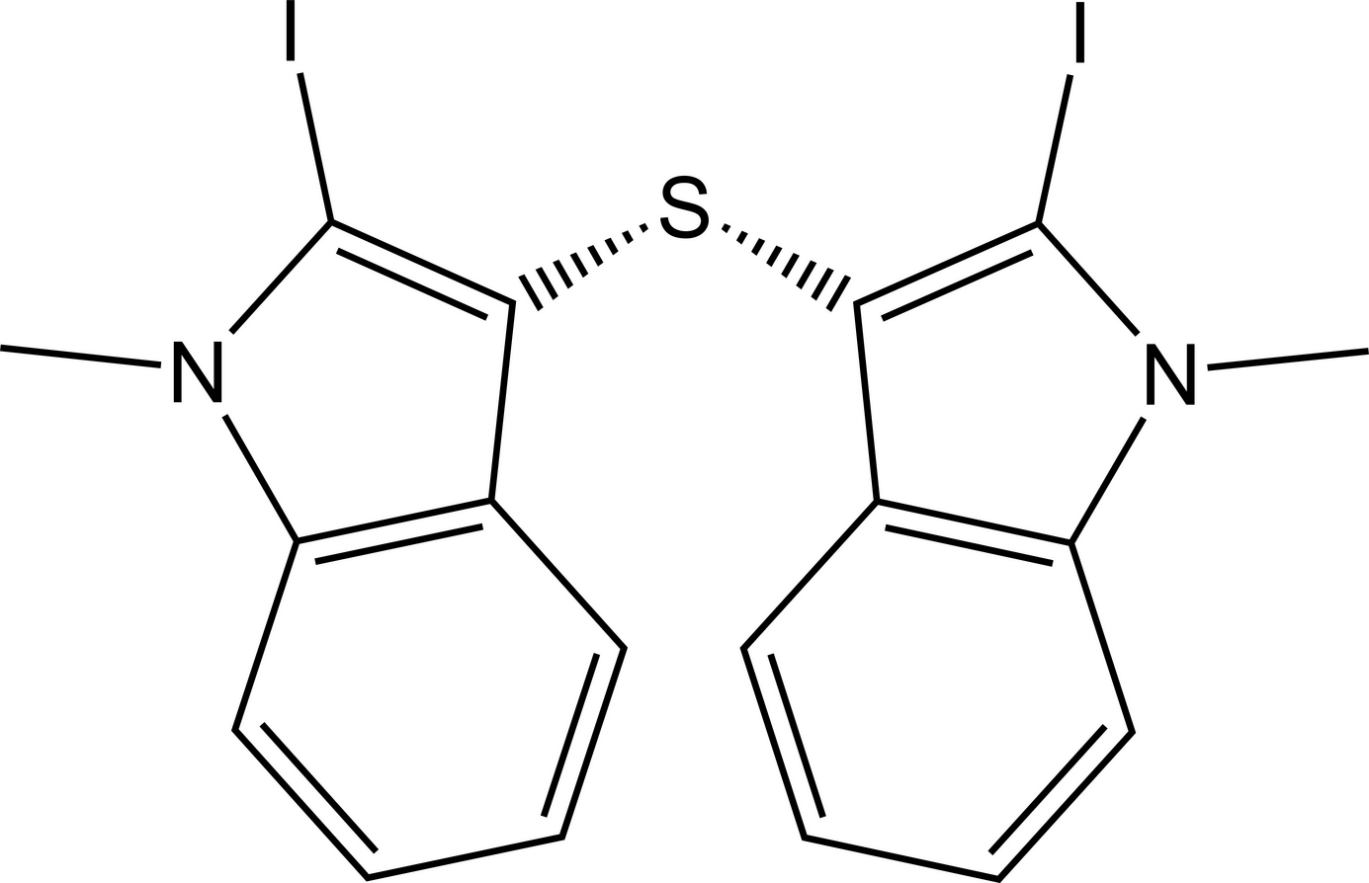


## 2. Structural commentary

**Figure 1:**
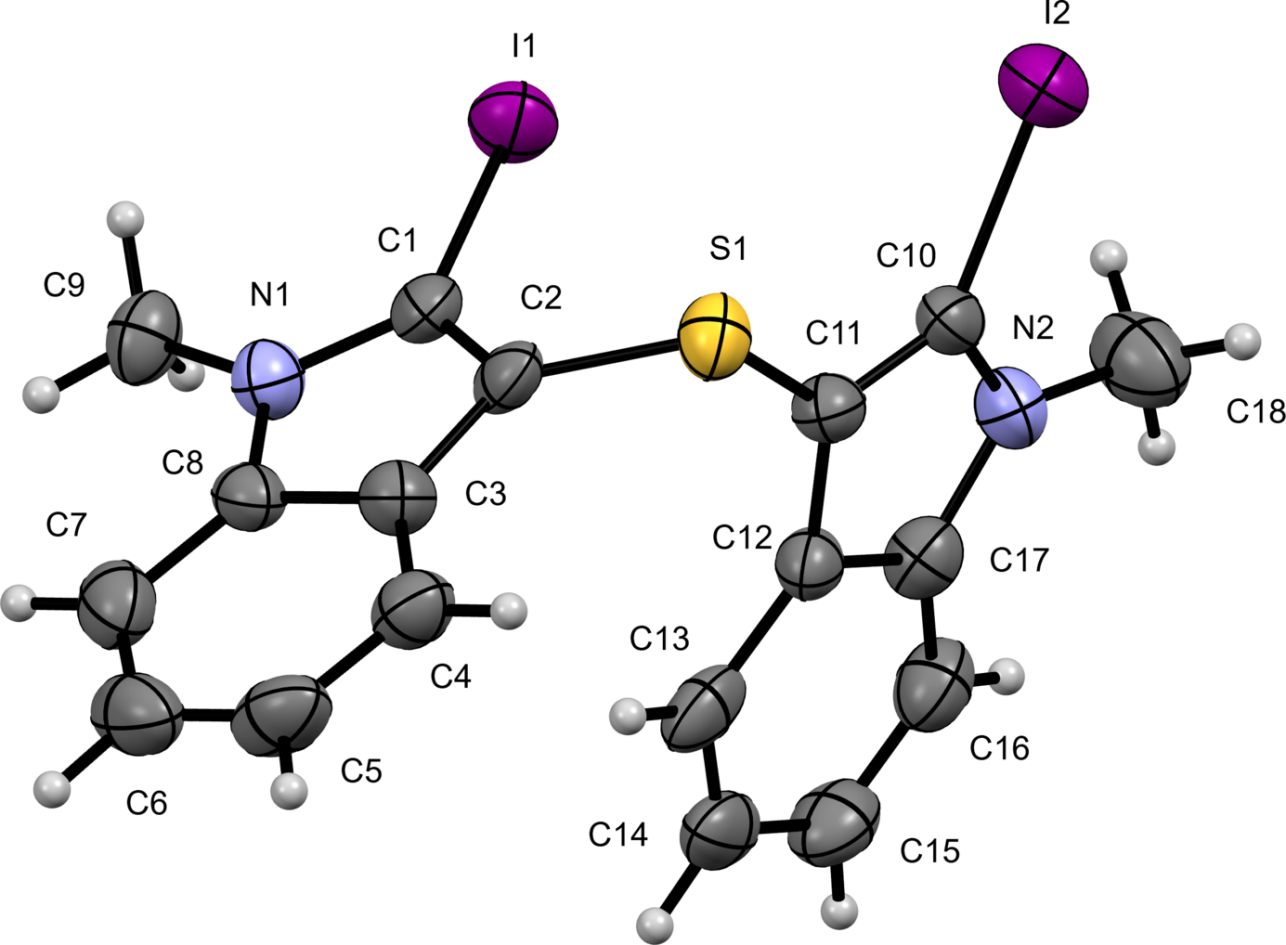
The mol­ecular structure of (**I**) with displacement ellipsoids drawn at the 50% probability level.

The mol­ecular structure of 3,3′-sulfanediylbis(2-iodo-1-methyl-1*H*-indole), (**I**), consists of two *N*-methyl-2-iodo­indole fragments linked through a thio­ether bridge at the 3-position (Fig. 1 ▸). The C—I bond lengths are similar [I1—C1 = 2.079 (12) Å and I2—C10 = 2.080 (9) Å]. The sulfur atom adopts a typical thio­ether geometry with S1—C2 = 1.761 (11) Å and S1—C11 = 1.770 (11) Å, and a C2—S1—C11 angle of 100.6 (5)°. The conformation about the S bridge is *gauche* on both sides, as shown by the torsion angles C11—S1—C2—C1 = −64.1 (11)° and C2—S1—C11—C12 = −48.3 (10)°. Both indole ring systems are essentially planar, with r.m.s. deviations of 0.012 Å for the N1/C1–C8 system and 0.017 Å for the N2/C10–C17 system. The two indole mean planes are strongly inclined, with an inter­planar angle (normal-to-normal) of 76.3 (3)°.

## 3. Supra­molecular features

**Figure 2:**
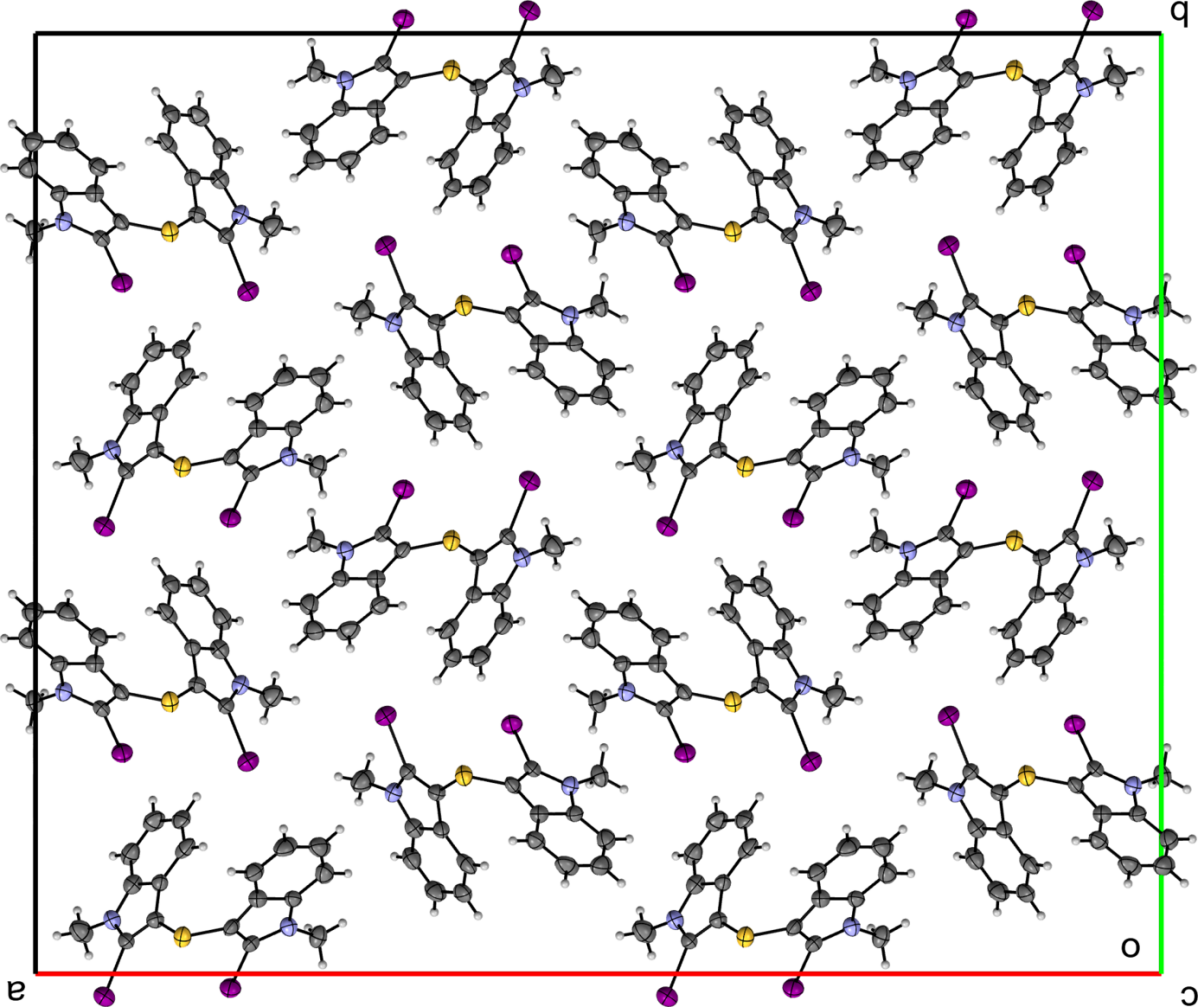
A view along the *c-*axis direction of the crystal packing of (**I**)

Compound (I) crystallizes in the ortho­rhom­bic space group *Fdd*2. In the crystal, the mol­ecule lacks classical hydrogen-bond donors and no significant hydrogen-bonding inter­actions are observed; likewise, no π–π stacking between indole rings is evident. The packing (Fig. 2 ▸) is dominated primarily by van der Waals contacts, but with shorter than van der Waals inter­molecular I⋯C contacts, C14⋯I1^i^ = 3.341 (12) Å and C5⋯I2^i^ = 3.433 (14) Å [symmetry code: (i) 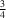 − *x*, 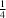 + *y*, −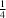 + *z*]. The mol­ecules pack in a herringbone pattern with each iodine substituent oriented toward the six-membered π-system of an indole ring in a neighbouring, symmetry-related mol­ecule, giving rise to I⋯π contacts. For I1, the perpendicular separation from the plane of the C12–C17 ring is 3.217 (13) Å and the iodine-to-centroid distance is I1⋯*Cg*(C12–C17)^i^ = 3.906 (5) Å; the C1—I1 vector makes an angle of 152.7 (4)° with the ring-plane normal and the C1—I1⋯*Cg*^i^ angle is 166.8 (3)°. Similarly, I2 lies 3.323 (14) Å from the plane of the C3–C8 ring with I2⋯*Cg*(C3–C8)^i^ = 3.999 (5) Å; the C10–I2 vector makes an angle of 25.0 (4)° with the ring-plane normal and the C10—I2⋯*Cg*^i^ angle is 171.2 (3)°. These I⋯π contacts link the mol­ecules into chains running parallel to the *b* axis.

## 4. Hirshfeld surface analysis

The inter­molecular inter­actions were further investigated by qu­anti­tative analysis of the Hirshfeld surface, and visualized with *Crystal Explorer 21.5* (Spackman *et al.*, 2021 ▸) and the two-dimensional fingerprint plots (McKinnon *et al.*, 2007 ▸). The shorter and longer contacts are indicated as red and blue spots, respectively, on the Hirshfeld surfaces, and contacts with distances approximately equal to the sum of the van der Waals radii are colored white. The function *d*_norm_ is a ratio enclosing the distances of any surface point to the nearest inter­ior (*d*_i_) and exterior (*d*_e_) atom and the van der Waals (vdW) radii of the atoms. The *d*_norm_ plots were mapped with a color scale between −0.19 a.u. (red) and +1.2 a.u. (blue).

**Figure 3:**
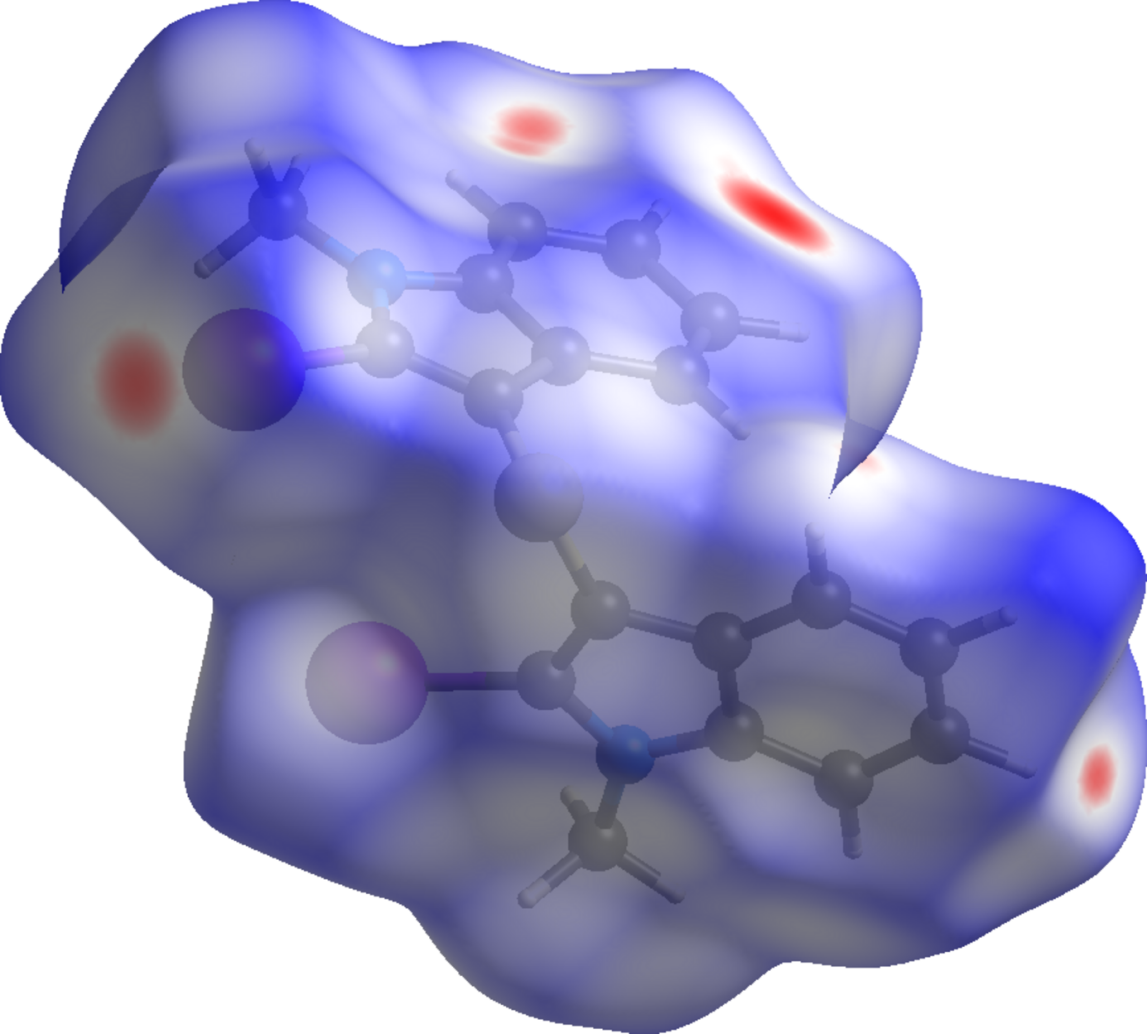
Hirshfeld surface for (**I**) mapped over *d*_norm_.

Fig. 3 ▸ shows the *d*_norm_ Hirshfeld surface of the title compound. The most intense red regions on the surface correspond to short inter­molecular contacts involving H⋯I and H⋯C inter­actions, indicating their importance in the crystal packing. No pronounced red–blue triangular features are observed on the shape-index surface, suggesting the absence of significant π–π stacking inter­actions. Analysis of the two-dimensional fingerprint plots reveals that H⋯H contacts make the largest contribution to the Hirshfeld surface (33.1%), highlighting the dominant role of van der Waals inter­actions in the packing. This is followed by H⋯C/C⋯H (25.5%) and H⋯I/I⋯H (24.4%) inter­actions, which together account for a substantial fraction of the inter­molecular contacts. Smaller contributions arise from C⋯I/I⋯C (5.2%), H⋯S/S⋯H (4.9%), and H⋯N/N⋯H (2.6%) contacts, while all remaining inter­actions contribute less than 2% individually, see Table 1 ▸.

## 5. Database survey

A search of the Cambridge Structural Database (CSD; website, accessed on 6 January 2026; Groom *et al.*, 2016 ▸) for structures related to the title compound, 3,3′-sulfanediylbis(2-iodo-1-methyl-1*H*-indole), returned nine relevant entries. Three structures feature the 3,3′-thio­ether-linked bis­(indole) motif: GETCAK, 1,1′-bis­(*t*-butyl­dimethyl­sil­yl)-3,3′-bis­(1*H*-indol-3-yl)sulfide (Shirani *et al.*, 2006 ▸), LOJTOX, 3,3′-sulfanediylbis(2-methyl-1*H*-indole) (Sharma *et al.*, 2024 ▸), and YIQPEV, 3,3′-sulfanediylbis(1-methyl-1*H*-indole) (Shibahara *et al.*, 2014 ▸); among these, YIQPEV is the closest analogue in terms of *N*-methyl­ation, but it lacks the 2-iodo substitution present in the title compound. The remaining entries comprise iodinated indole derivatives with differing substitution patterns and functionalization, including JEVQOR, 2,3-di­iodo-1-(phenyl­sulfon­yl)-1*H*-indole (Rinderspacher *et al.*, 2007 ▸), KAGQIV, 2-iodo-1-phenyl-1*H*-indole (Messaoud *et al.*, 2015 ▸), iodo­indole benzamide derivatives KAGGEJ and KAGGIN (Kim *et al.*, 2025 ▸), and 2-iodo-3-[(tri­fluoro­meth­yl)selan­yl]indole derivatives KOYFAJ and KOYFIR (Huang *et al.*, 2024 ▸).

## 6. Synthesis and crystallization

Di-*tert*-butyl di­sulfide (200 mg, 1.12 mmol) and *N*-methyl indole (147 mg, 1.12 mmol) were placed in a round-bottomed flask along with 2 mL of DMSO. The mixture was flushed with argon and iodine (710 mg, 2.8 mmol) and one drop of DBU (1,8-diazabicyclo[5.4.0]undec-7-ene) were added successively. The reaction mixture was then allowed to stir at room temperature for 12 h. After satisfactory conversion as indicated on TLC (10% ethyl acetate: hexa­ne), the reaction mixture was washed with dilute sodium thio­sulfate and the product was extracted in ethyl acetate. The crude product was subjected to purification using column chromatography to obtain a white solid (200 mg, 66%). Crystals for X-ray analysis were obtained by slow evaporation from ethyl acetate solution at room temperature.

Spectroscopic data: ^1^H NMR (400 MHz, CDCl_3_) δ = 7.75 (*d*, *J* = 6.3 Hz, 2H), 7.27 (*m*, 2H), 7.15 (*m*, 4H), 3.78 (*s*, 6H). ^13^C NMR (101 MHz, CDCl_3_) δ = 138.8, 129.6, 122.2, 120.1, 119.9, 112.6, 109.7, 97.12, 35.1.

## 7. Refinement

Crystal data, data collection and structure refinement details are summarized in Table 2 ▸. H atoms were positioned geometrically (C—H = 0.93–0.96 Å) and refined as riding with *U*_iso_(H) = 1.2*U*_eq_(C) or 1.5*U*_eq_(Cmeth­yl).

## Supplementary Material

Crystal structure: contains datablock(s) I. DOI: 10.1107/S2056989026002872/ee2026sup1.cif

Structure factors: contains datablock(s) I. DOI: 10.1107/S2056989026002872/ee2026Isup2.hkl

CCDC reference: 2539020

Additional supporting information:  crystallographic information; 3D view; checkCIF report

## Figures and Tables

**Table 1:** Contributions of selected inter­molecular contacts (%) to the Hirshfeld surfaces of (I)

Contact	(%)
H⋯H	33.1
H⋯C/C⋯H	25.5
H⋯I/I⋯H	24.4
C⋯I/I⋯C	5.2
H⋯S/S⋯H	4.9
H⋯N/N⋯H	2.6
I⋯S/S⋯I	1.7
C⋯S/S⋯C	1.4
S⋯N/N⋯S	0.9
I⋯I	0.2

**Table 2:** Experimental details

Crystal data	
Chemical formula	C_18_H_14_I_2_N_2_S
*M*_r_	544.17
Crystal system, space group	Orthorhombic, *Fdd*2
Temperature (K)	298
*a*, *b*, *c* (Å)	37.8416 (4), 31.6009 (3), 5.94404 (5)
*V* (Å^3^)	7108.05 (11)
*Z*	16
Radiation type	Cu *K*α
μ (mm^−1^)	28.89
Crystal size (mm)	0.15 × 0.05 × 0.02

Data collection	
Diffractometer	XtaLAB Synergy, Single source at home/near, HyPix3000
Absorption correction	Multi-scan (*CrysAlis PRO*; Rigaku OD, 2023 ▸)
*T*_min_, *T*_max_	0.401, 1.000
No. of measured, independent and observed [*I* > 2σ(*I*)] reflections	9127, 2434, 2352
*R*_int_	0.099
(sin θ/λ)_max_ (Å^−1^)	0.608

Refinement	
*R*[*F*^2^ > 2σ(*F*^2^)], *wR*(*F*^2^), *S*	0.052, 0.146, 1.18
No. of reflections	2434
No. of parameters	210
No. of restraints	1
H-atom treatment	H-atom parameters constrained
Δρ_max_, Δρ_min_ (e Å^−3^)	0.90, −1.60
Absolute structure	Flack *x* determined using 558 quotients [(*I*^+^)−(*I*^−^)]/[(*I*^+^)+(*I*^−^)] (Parsons *et al.*, 2013 ▸)
Absolute structure parameter	−0.034 (16)

Computer programs: *CrysAlis PRO* (Rigaku OD, 2023 ▸), *SHELXL2018/3* (Sheldrick, 2015 ▸) and *OLEX2* (Dolomanov *et al.*, 2009 ▸).

## supplementary crystallographic information

### 2-Iodo-3-[(2-iodo-1-methyl-1H-indol-3-yl)sulfanyl]-1-methyl-1H-indole Crystal data

**Table:**

C_18_H_14_I_2_N_2_S	*D*_x_ = 2.034 Mg m^−^^3^
*M**_r_* = 544.17	Cu *K*α radiation, λ = 1.54184 Å
Orthorhombic, *F**d**d*2	Cell parameters from 7695 reflections
*a* = 37.8416 (4) Å	θ = 2.8–69.4°
*b* = 31.6009 (3) Å	µ = 28.89 mm^−^^1^
*c* = 5.94404 (5) Å	*T* = 298 K
*V* = 7108.05 (11) Å^3^	Needle, clear colourless
*Z* = 16	0.15 × 0.05 × 0.02 mm
*F*(000) = 4128	

### 2-Iodo-3-[(2-iodo-1-methyl-1H-indol-3-yl)sulfanyl]-1-methyl-1H-indole Data collection

**Table:**

XtaLAB Synergy, Single source at home/near, HyPix3000 diffractometer	2434 independent reflections
Radiation source: micro-focus sealed X-ray tube, PhotonJet (Cu) X-ray Source	2352 reflections with *I* > 2σ(*I*)
Mirror monochromator	*R*_int_ = 0.099
Detector resolution: 10.0000 pixels mm^-1^	θ_max_ = 69.6°, θ_min_ = 3.7°
ω scans	*h* = −45→39
Absorption correction: multi-scan (CrysAlisPro; Rigaku OD, 2023)	*k* = −38→38
*T*_min_ = 0.401, *T*_max_ = 1.000	*l* = −7→5
9127 measured reflections	

### 2-Iodo-3-[(2-iodo-1-methyl-1H-indol-3-yl)sulfanyl]-1-methyl-1H-indole Refinement

**Table:**

Refinement on *F*^2^	Hydrogen site location: inferred from neighbouring sites
Least-squares matrix: full	H-atom parameters constrained
*R*[*F*^2^ > 2σ(*F*^2^)] = 0.052	*w* = 1/[σ^2^(*F*_o_^2^) + (0.1145*P*)^2^] where *P* = (*F*_o_^2^ + 2*F*_c_^2^)/3
*wR*(*F*^2^) = 0.146	(Δ/σ)_max_ = 0.001
*S* = 1.18	Δρ_max_ = 0.90 e Å^−^^3^
2434 reflections	Δρ_min_ = −1.60 e Å^−^^3^
210 parameters	Absolute structure: Flack *x* determined using 558 quotients [(*I*^+^)-(*I*^-^)]/[(*I*^+^)+(*I*^-^)](Parsons *et al.*, 2013)
1 restraint	Absolute structure parameter: −0.034 (16)

### 2-Iodo-3-[(2-iodo-1-methyl-1H-indol-3-yl)sulfanyl]-1-methyl-1H-indole Special details

**Table:**

Geometry. All esds (except the esd in the dihedral angle between two l.s. planes) are estimated using the full covariance matrix. The cell esds are taken into account individually in the estimation of esds in distances, angles and torsion angles; correlations between esds in cell parameters are only used when they are defined by crystal symmetry. An approximate (isotropic) treatment of cell esds is used for estimating esds involving l.s. planes.

### 2-Iodo-3-[(2-iodo-1-methyl-1H-indol-3-yl)sulfanyl]-1-methyl-1H-indole Fractional atomic coordinates and isotropic or equivalent isotropic displacement parameters (Å^2^)

**Table:**

	*x*	*y*	*z*	*U*_iso_*/*U*_eq_	
I1	0.32692 (2)	0.48490 (2)	0.93756 (14)	0.0508 (3)	
I2	0.43892 (2)	0.47566 (2)	0.71981 (17)	0.0578 (3)	
S1	0.36946 (6)	0.53925 (8)	0.4606 (5)	0.0439 (6)	
C5	0.2780 (3)	0.6351 (4)	0.230 (3)	0.061 (3)	
H5	0.279642	0.654130	0.111082	0.073*	
C14	0.3706 (3)	0.6641 (3)	0.938 (3)	0.055 (3)	
H14	0.356860	0.688280	0.920913	0.066*	
C7	0.2432 (3)	0.6070 (4)	0.538 (3)	0.052 (3)	
H7	0.222570	0.606600	0.622775	0.062*	
C16	0.4158 (3)	0.6273 (4)	1.158 (2)	0.052 (3)	
H16	0.431069	0.625179	1.280030	0.063*	
C9	0.2490 (3)	0.5395 (4)	0.926 (2)	0.047 (2)	
H9A	0.245021	0.509541	0.927082	0.071*	
H9B	0.227240	0.553922	0.894335	0.071*	
H9C	0.257864	0.548363	1.069472	0.071*	
C6	0.2476 (4)	0.6347 (4)	0.360 (3)	0.061 (3)	
H6	0.229574	0.653719	0.326893	0.073*	
C12	0.3896 (2)	0.5966 (3)	0.815 (2)	0.037 (2)	
C13	0.3671 (3)	0.6333 (4)	0.786 (2)	0.050 (3)	
H13	0.351147	0.635244	0.667507	0.060*	
C18	0.4594 (4)	0.5450 (5)	1.140 (3)	0.067 (4)	
H18A	0.480997	0.540454	1.058117	0.101*	
H18B	0.452750	0.519378	1.215169	0.101*	
H18C	0.462896	0.567063	1.248555	0.101*	
N1	0.2752 (2)	0.5500 (3)	0.7496 (16)	0.0410 (18)	
C17	0.4136 (3)	0.5951 (4)	0.998 (2)	0.043 (2)	
C15	0.3947 (3)	0.6622 (4)	1.128 (2)	0.055 (3)	
H15	0.395848	0.684538	1.229133	0.066*	
C10	0.4193 (2)	0.5350 (3)	0.805 (2)	0.039 (2)	
C11	0.3936 (2)	0.5572 (3)	0.697 (2)	0.039 (2)	
C4	0.3058 (3)	0.6082 (3)	0.272 (2)	0.047 (3)	
H4	0.326016	0.608604	0.182858	0.057*	
C1	0.3086 (2)	0.5320 (3)	0.723 (2)	0.040 (2)	
N2	0.4320 (2)	0.5572 (3)	0.986 (2)	0.047 (2)	
C3	0.3025 (3)	0.5800 (3)	0.454 (2)	0.042 (2)	
C2	0.3259 (2)	0.5495 (3)	0.550 (2)	0.039 (2)	
C8	0.2712 (3)	0.5797 (3)	0.5853 (19)	0.039 (2)	

### 2-Iodo-3-[(2-iodo-1-methyl-1H-indol-3-yl)sulfanyl]-1-methyl-1H-indole Atomic displacement parameters (Å^2^)

**Table:**

	*U*^11^	*U*^22^	*U*^33^	*U*^12^	*U*^13^	*U*^23^
I1	0.0529 (4)	0.0460 (4)	0.0535 (5)	0.0002 (3)	−0.0029 (3)	0.0119 (3)
I2	0.0527 (4)	0.0459 (4)	0.0748 (6)	0.0076 (3)	0.0014 (4)	−0.0028 (4)
S1	0.0357 (10)	0.0523 (13)	0.0437 (14)	−0.0027 (10)	−0.0002 (10)	−0.0062 (12)
C5	0.067 (7)	0.046 (5)	0.069 (8)	−0.012 (5)	−0.021 (7)	0.020 (6)
C14	0.044 (5)	0.041 (5)	0.079 (9)	−0.008 (4)	0.023 (7)	0.002 (6)
C7	0.041 (5)	0.054 (6)	0.061 (7)	−0.001 (5)	−0.006 (5)	−0.009 (6)
C16	0.045 (5)	0.060 (6)	0.052 (7)	−0.012 (5)	0.001 (5)	−0.010 (6)
C9	0.040 (4)	0.060 (6)	0.042 (6)	−0.007 (5)	0.005 (5)	−0.003 (5)
C6	0.059 (6)	0.045 (6)	0.080 (10)	0.004 (5)	−0.028 (7)	−0.002 (6)
C12	0.031 (4)	0.037 (4)	0.043 (5)	−0.002 (3)	0.003 (4)	−0.006 (4)
C13	0.047 (6)	0.053 (6)	0.051 (7)	−0.023 (5)	0.017 (5)	−0.011 (5)
C18	0.067 (8)	0.064 (8)	0.071 (9)	0.014 (6)	−0.018 (8)	−0.009 (7)
N1	0.037 (4)	0.043 (4)	0.043 (5)	0.001 (3)	0.004 (4)	−0.001 (4)
C17	0.042 (4)	0.045 (5)	0.043 (6)	−0.006 (4)	0.000 (5)	0.001 (5)
C15	0.058 (6)	0.051 (6)	0.057 (8)	−0.012 (5)	0.004 (6)	−0.016 (6)
C10	0.033 (4)	0.031 (4)	0.054 (7)	0.002 (4)	0.007 (4)	0.001 (4)
C11	0.037 (4)	0.033 (4)	0.046 (6)	−0.002 (4)	0.002 (5)	−0.004 (4)
C4	0.048 (5)	0.042 (5)	0.051 (7)	−0.008 (5)	−0.004 (5)	0.007 (5)
C1	0.033 (4)	0.033 (4)	0.053 (6)	−0.007 (3)	−0.001 (5)	−0.004 (5)
N2	0.037 (4)	0.044 (4)	0.059 (6)	0.005 (4)	−0.006 (4)	0.001 (5)
C3	0.040 (4)	0.037 (4)	0.048 (6)	0.000 (4)	−0.001 (5)	0.000 (5)
C2	0.033 (4)	0.037 (5)	0.047 (6)	−0.012 (4)	−0.010 (4)	0.005 (5)
C8	0.038 (4)	0.031 (4)	0.047 (5)	0.002 (4)	−0.006 (4)	−0.004 (4)

### 2-Iodo-3-[(2-iodo-1-methyl-1H-indol-3-yl)sulfanyl]-1-methyl-1H-indole Geometric parameters (Å, º)

**Table:**

I1—C1	2.079 (12)	C6—H6	0.9300
I2—C10	2.080 (9)	C12—C13	1.448 (17)
S1—C11	1.770 (11)	C12—C17	1.418 (16)
S1—C2	1.761 (11)	C12—C11	1.436 (13)
C5—H5	0.9300	C13—H13	0.9300
C5—C6	1.39 (2)	C18—H18A	0.9600
C5—C4	1.375 (18)	C18—H18B	0.9600
C14—H14	0.9300	C18—H18C	0.9600
C14—C13	1.336 (17)	C18—N2	1.435 (17)
C14—C15	1.452 (19)	N1—C1	1.393 (12)
C7—H7	0.9300	N1—C8	1.364 (14)
C7—C6	1.38 (2)	C17—N2	1.390 (14)
C7—C8	1.394 (15)	C15—H15	0.9300
C16—H16	0.9300	C10—C11	1.361 (14)
C16—C17	1.392 (16)	C10—N2	1.369 (16)
C16—C15	1.373 (19)	C4—H4	0.9300
C9—H9A	0.9600	C4—C3	1.409 (16)
C9—H9B	0.9600	C1—C2	1.342 (17)
C9—H9C	0.9600	C3—C2	1.428 (14)
C9—N1	1.476 (14)	C3—C8	1.417 (15)
			
C2—S1—C11	100.6 (5)	C1—N1—C9	126.6 (9)
C6—C5—H5	119.0	C8—N1—C9	126.0 (9)
C4—C5—H5	119.0	C8—N1—C1	107.4 (9)
C4—C5—C6	121.9 (12)	C16—C17—C12	122.7 (10)
C13—C14—H14	118.1	N2—C17—C16	129.3 (11)
C13—C14—C15	123.8 (12)	N2—C17—C12	108.0 (10)
C15—C14—H14	118.1	C14—C15—H15	120.0
C6—C7—H7	121.5	C16—C15—C14	120.0 (11)
C6—C7—C8	117.0 (12)	C16—C15—H15	120.0
C8—C7—H7	121.5	C11—C10—I2	127.2 (9)
C17—C16—H16	121.3	C11—C10—N2	111.0 (9)
C15—C16—H16	121.3	N2—C10—I2	121.9 (7)
C15—C16—C17	117.5 (12)	C12—C11—S1	127.6 (8)
H9A—C9—H9B	109.5	C10—C11—S1	125.4 (8)
H9A—C9—H9C	109.5	C10—C11—C12	107.0 (10)
H9B—C9—H9C	109.5	C5—C4—H4	121.2
N1—C9—H9A	109.5	C5—C4—C3	117.5 (12)
N1—C9—H9B	109.5	C3—C4—H4	121.2
N1—C9—H9C	109.5	N1—C1—I1	121.7 (8)
C5—C6—H6	119.0	C2—C1—I1	127.0 (7)
C7—C6—C5	122.1 (11)	C2—C1—N1	111.3 (10)
C7—C6—H6	119.0	C17—N2—C18	124.2 (11)
C17—C12—C13	119.5 (10)	C10—N2—C18	128.0 (9)
C17—C12—C11	106.2 (9)	C10—N2—C17	107.8 (9)
C11—C12—C13	134.3 (10)	C4—C3—C2	132.7 (11)
C14—C13—C12	116.5 (13)	C4—C3—C8	120.1 (10)
C14—C13—H13	121.8	C8—C3—C2	107.2 (10)
C12—C13—H13	121.8	C1—C2—S1	127.9 (8)
H18A—C18—H18B	109.5	C1—C2—C3	106.3 (9)
H18A—C18—H18C	109.5	C3—C2—S1	125.8 (9)
H18B—C18—H18C	109.5	C7—C8—C3	121.4 (10)
N2—C18—H18A	109.5	N1—C8—C7	130.8 (11)
N2—C18—H18B	109.5	N1—C8—C3	107.8 (9)
N2—C18—H18C	109.5		
			
I1—C1—C2—S1	−2.6 (15)	C17—C12—C11—C10	−1.5 (12)
I1—C1—C2—C3	179.6 (8)	C15—C14—C13—C12	−1.3 (16)
I2—C10—C11—S1	2.4 (15)	C15—C16—C17—C12	−2.7 (17)
I2—C10—C11—C12	−179.0 (7)	C15—C16—C17—N2	179.0 (11)
I2—C10—N2—C18	0.6 (18)	C11—S1—C2—C1	−64.1 (11)
I2—C10—N2—C17	−179.7 (7)	C11—S1—C2—C3	113.3 (10)
C5—C4—C3—C2	−177.0 (12)	C11—C12—C13—C14	178.3 (11)
C5—C4—C3—C8	0.6 (16)	C11—C12—C17—C16	−176.7 (10)
C16—C17—N2—C18	−3 (2)	C11—C12—C17—N2	1.9 (12)
C16—C17—N2—C10	176.9 (11)	C11—C10—N2—C18	−179.0 (13)
C9—N1—C1—I1	0.9 (14)	C11—C10—N2—C17	0.7 (13)
C9—N1—C1—C2	−178.3 (10)	C4—C5—C6—C7	−1 (2)
C9—N1—C8—C7	−0.2 (18)	C4—C3—C2—S1	1.5 (18)
C9—N1—C8—C3	179.3 (10)	C4—C3—C2—C1	179.4 (11)
C6—C5—C4—C3	−0.3 (19)	C4—C3—C8—C7	0.1 (16)
C6—C7—C8—N1	178.5 (11)	C4—C3—C8—N1	−179.5 (10)
C6—C7—C8—C3	−1.0 (17)	C1—N1—C8—C7	−179.0 (11)
C12—C17—N2—C18	178.1 (12)	C1—N1—C8—C3	0.6 (11)
C12—C17—N2—C10	−1.6 (12)	N2—C10—C11—S1	−178.0 (8)
C13—C14—C15—C16	0.7 (18)	N2—C10—C11—C12	0.6 (12)
C13—C12—C17—C16	2.1 (16)	C2—S1—C11—C12	−48.3 (10)
C13—C12—C17—N2	−179.3 (9)	C2—S1—C11—C10	130.0 (10)
C13—C12—C11—S1	−1.5 (18)	C2—C3—C8—C7	178.3 (10)
C13—C12—C11—C10	180.0 (11)	C2—C3—C8—N1	−1.3 (12)
N1—C1—C2—S1	176.6 (8)	C8—C7—C6—C5	1.3 (19)
N1—C1—C2—C3	−1.3 (13)	C8—N1—C1—I1	179.7 (7)
C17—C16—C15—C14	1.3 (17)	C8—N1—C1—C2	0.5 (12)
C17—C12—C13—C14	−0.1 (15)	C8—C3—C2—S1	−176.3 (8)
C17—C12—C11—S1	177.0 (8)	C8—C3—C2—C1	1.6 (12)
